# First reference on reproductive biology of *Butis koilomatodon* in Mekong Delta, Vietnam

**DOI:** 10.1186/s40850-021-00072-y

**Published:** 2021-04-21

**Authors:** Quang M. Dinh, Tran T. H. Lam, Ton H. D. Nguyen, Thanh M. Nguyen, Tien T. K. Nguyen, Nam T. Nguyen

**Affiliations:** 1 Department of Biology, School of Education, Can Tho University, Xuan Khanh Ward, Ninh Kieu District, Can Tho, 900000 Vietnam; 2 PhD student at Biotechnology Research and Development Institute, Can Tho University, Xuan Khanh Ward, Ninh Kieu District, Can Tho, 900000 Vietnam; 3 Department of Biotechnology, Faculty of Agriculture and Fishery, University of Cuu Long, Phu Quoi Ward, Long Ho District, Vinh Long, 890000 Vietnam; 4 An Khanh High School, An Khanh Ward, Ninh Kieu District, Can Tho, 900000 Vietnam; 5 Faculty of Biology, VNU University of Science, Vietnam National University, 334 Nguyen Trai, Thanh Xuan District, Ha Noi, 100000 Vietnam; 6 Biological Museum, VNU University of Science, Vietnam National University, 19 Le Thanh Tong, Hoan Kiem District, Ha Noi, 100000 Vietnam

**Keywords:** Fecundity, Gonadosomatic index, Length at first mature, Multi-spawner

## Abstract

**Background:**

The key to fishery management is knowing the appropriate reproductive strategies of the targeted fish. For most gobiid species, the iteroparous pattern is dominant compared to semelparity. Albeit *Butis koilomatodon* plays an important role in the Mekong Delta’s food supply, its reproductive biological data have not been known. Hence, this study was conducted to provide new fundamental knowledge of reproductive traits of *Butis koilomatodon* in the Mekong Delta.

**Results:**

A total of 1314 individuals (903 males and 411 females) were monthly collected by bottom gill nets from July 2019 to June 2020 at six sampling sites along estuarial and coastal regions, from Tra Vinh to Ca Mau provinces, southern of Vietnam. pH and salinity of these six sampling sites are 7.72–7.93 pH and 11.17–26.17‰, respectively. The pH varies with sites, but not seasons; whereas a reverse case is found in salinity. Different types of oocytes are found in histological specimens of ovaries prove that *B. koilomatodon* is a multi-spawner. The gonadosomatic index value, together with the monthly presence of mature ovaries reveal that this species spawns throughout the year. The length at first mature male *Butis koilomatodon* (5.1–8.6 cm) is higher than that of females (4.8–6.7 cm), except in Hoa Binh and Dong Hai. Batch fecundity (3085 to 32,087 eggs/female) increases with fish weight (1.48–12.30 g) and length (4.8–9.0 cm) due to high determination values (*r*^*2*^ > 0.6).

**Conclusion:**

Knowledge of reproductive traits gained from this study was a reference source for future studies and helped manage this species’ resources.

**Supplementary Information:**

The online version contains supplementary material available at 10.1186/s40850-021-00072-y.

## Background

Miller et al. [1] state that *Butis koilomatodon* (Bleeker, 1849) (Perciformes: Eleotridae) is native to the Indo-Pacific estuarial region and adapts well to a wide range of salinity from 3.8‰ to 37.0‰, showing that this fish species becomes a potential candidate for aquaculture in these mangrove regions. In Vietnam, *Butis* genus consists of five species *Butis butis, Butis amboinensis, Butis gymnopomus, Butis koilomatodon,* and *Butis humeralis* [2]. The species *Butis koilomatodon* is recorded mainly in seashores of south-western provinces of Vietnam (Duyen Hai, Tra Vinh province; Cu Lao Dung and Tran De, Soc Trang province; Hoa Binh and Dong Hai, Bac Lieu province; and Dam Doi, Ca Mau province) [3, 4]. Nevertheless, this species faces overexploitation by many destructive types of machinery [5], which seriously soaked up this species’ natural yield. Understanding their reproductive biology is essential as it presents a suitable solution for the conservation and recruitment of this goby.

The two main fish reproductive patterns are semelparity and iteroparity, consisting of synchronism and asynchronism [6]. For most gobiid species, the iteroparous pattern is dominant [7, 8]. In most cases, male gobies care for the eggs after fertilization, whereas females rarely take part in parental care [9, 10], namely *Neogobius melanostomus* [11] and *Periophthalmus minutus* [12, 13]. In other cases, the females do take care of the eggs like *Awaous guamensis* [14]. In gobies, there are five to several hundred eggs released by females on vegetation or a substrate (bottom surface), and then males fertilized. For gobies living in estuaries, the lunar cycle plays a vital role in spawning behavior and larval recruitment [9, 10]. There is a close relationship between fish stock management and fish size at first maturity and batch fecundity [15, 16]. The information related to a commercial goby *Butis koilomatodon* reproduction is still insufficient so that further knowledge in its reproductive traits is necessary to conserve this gobiid population in Mekong Delta. Consequently, the present research aimed to determine spawning pattern and season, estimate the size at first mature and batch fecundity of *Butis koilomatodon* in the Mekong Delta, Vietnam.

## Methods

### Study site and fish collection

**Fig. 1:**
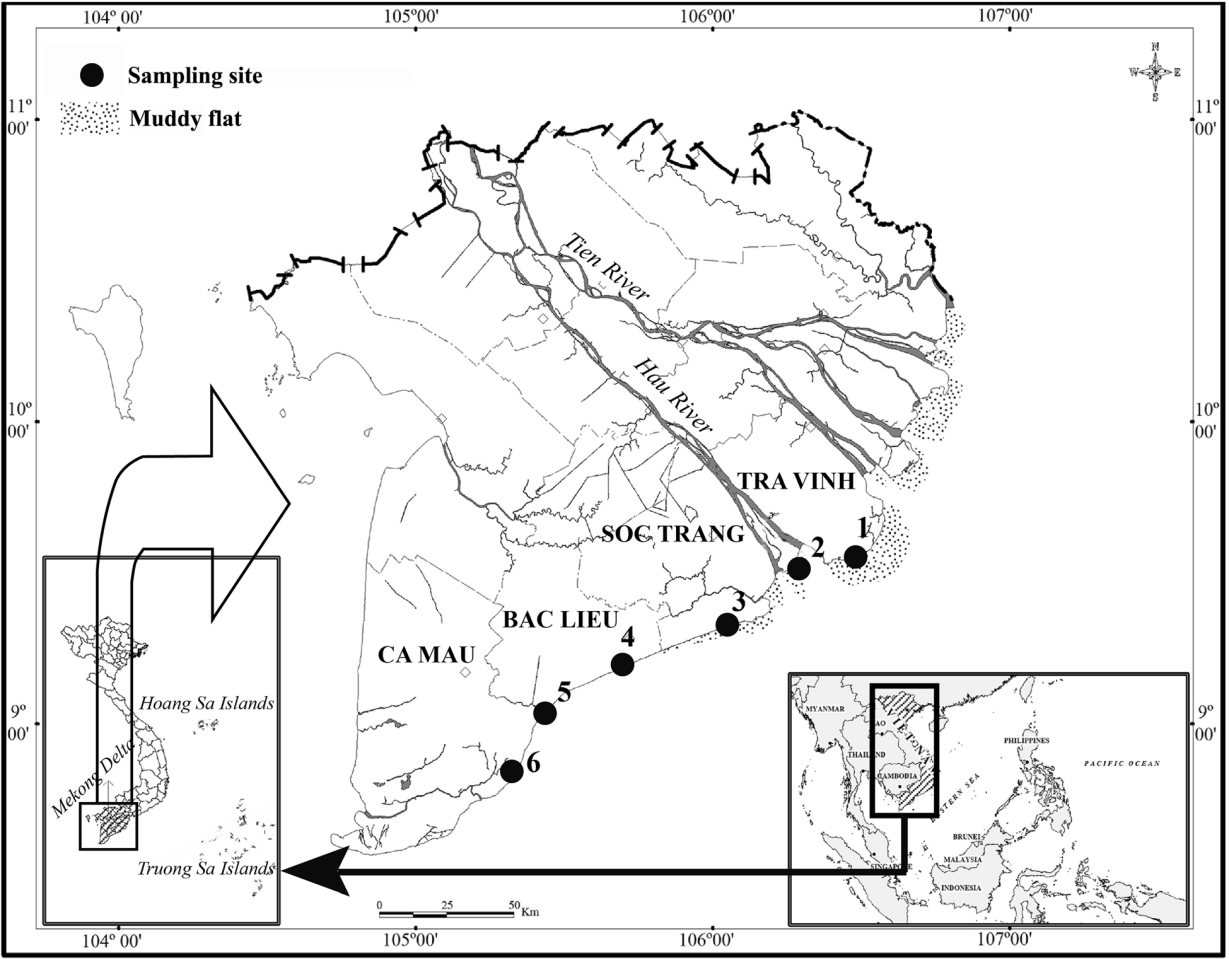
The sampling map in the Mekong Delta (•: Sampling area; 1: Duyen Hai, Tra Vinh, 2: Cu Lao Dung, Soc Trang, 3: Tran De, Soc Trang, 4: Hoa Binh, Bac Lieu, 5: Dong Hai, Bac Lieu, and 6: Dam Doi, Ca Mau). This map has been modified from the figure of Tran and Dinh [55] with permission

This study was conducted for one year, from July 2019 to June 2020, in estuarine and coastal regions including Duyen Hai (Tra Vinh province), Cu Lao Dung and Tran De (Soc Trang province), Hoa Binh and Dong Hai (Bac Lieu province) and Dam Doi (Ca Mau province) (Fig. 1). The tide type is semi-diurnal. There are only two seasons in these provinces regarding monsoon climate: from January to May–the dry season and from June to December–the wet season. The rainfall is highest in the wet season with approximately 400 mm, whereas it is rarely rain in the dry season. 27 °C is the average temperature [17]. *Avicennia marina* and *Sonneratia caseolaris* were indicator plants for these mangrove lands [18]. The pH and salinity of water were monthly recorded in each sampling site using a thermometer (Model: HI98127) and a refractometer (Model: 950.0100 PPT-ATC), respectively.

Samples of *Butis koilomatodon* were caught by using bottom gill nets. The net (5 m long, 1.5 cm mesh size in the cod end, and 2.5 cm in the mouth) was set continuously for 48 h. Then, fish samples were collected when the tide receded. Next, fish specimens were classified using their external morphology, e.g., beige body color, serrated snout and several dark bands were usually four to seven on *Butis koilomatodon* body that was a unique character to separate it from congeners [19]. Finally, fish samples were anesthetized by Tricaine Methanesulfonate with a ratio of 10 g/L using tap water before storing in 5% formalin buffer and transporting to the laboratory.

### Fish analysis

The total length (TL) and weight (W) of specimens in the laboratory were determined to the nearest 0.1 cm and 0.01 g, respectively. Based on the urogenital papilla’s external shape, the fish sex was determined, e.g., males had the narrow genital papilla while females had round one [3]. After dissection, ovaries and testes were separated from the fish body. Fish’s six maturation stages were visually classified according to the description of Vesey and Langford [20] on *Gobius niger*. Tissue samples (*n* = 40) of ovaries and testes represented four developmental stages from I to IV (five histological samples per stage) were stained and examined by following the method of Carleton et al. [21]. Samples of gonad glands (120 ovaries and 120 testes) corresponding to stages from I to IV were observed under the magnifier (Motic) and measured diameters by the scale of Motic Images Pro Plus 2.0 software. As identifying oocyte and spermatocyte’s developmental stages, the gamete term described by Yamamoto [22] and Yamazaki [23] was used.

The gonadosomatic index (*GSI*) was calculated by the following formula Sturm [24]: *GSI = 100 × (G/W)*, where *G* is gonad weight (0.1 mg) and *W* is total fish body weight (0.01 g). A combination of *GSI*’s analysis and gonad’s frequent occurrence was used to determine the fish spawning season [25, 26].

The logistic curve was used to estimate fish length at first maturity (*L*_*m*_) for each sampling sies according to the equation: *P = 1/(1 + exp[−r × (TL-L*_*m*_*)])*, where *P* is the proportion of mature individuals in a length class; *TL* is fish total length, and *r* is a model parameter [27].

Batch fecundity (*F*: number of oocytes released per spawning) was estimated gravimetrically [28]. In each sampling site, a total of 30 fish samples with ovaries in stages IV were used for oocyte counting. Each ovary was sectioned into three slices (sub-samples) in around 1-mm thickness at the two ends and in the middle. After weighing, each slice was put into a petri dish with tap water to separate oocytes using a spear-shaped needle. All the maturing oocytes were counted under the magnifier (Motic). Batch fecundity was determined from the eq. *F = (n × G)/g*, where *n* is the number of oocytes in sub-sample; *g* is the weight of sub-sample; and *G* is the ovarian weight [29].

### Data analysis

The normal distribution of pH, salinity, *GSI* and fecundity was tested by Shapiro-Wilk test [30]. T-test with Levene’s test for equality of variances was used to confirm whether *GSI* varied with gender and season in each sampling site if *GSI* dispayed normal distribution, whereas Mann-Whitney Test was used in the reverse case. The Levene’s test was also used to quantify the equality of variances of pH, salinity, GSI, and fecundity among six sampling sites and twelve months. If the variances of these three dependent factors were equal, one-way ANOVA with Tukey Post Hoc was used, whereas one-way ANOVA with Tamhane’s T2 was applied in the reverse case. Indeed, one-way ANOVA with Tukey Post Hoc was used to confirm whether the differences in pH and salinity among twelve months and six sampling sites exist, whereas one-way ANOVA with Tamhane’s T2 was used when the temporal and spatial variation of *GSI* was qualified. One-way ANOVA with Tukey Post Hoc was used to examine the effects of spatial difference on the batch fecundity, whereas one-way ANOVA with Tamhane’s T2 was applied in the reverse case. However, Kruskal-Wallis Test was used to confirm whether pH, salinity, GSI varied among six sampling sites and twelve months if these three dependent variables did not display normal distribution. Likewise, the variation of fecundity with sampling sites was qualified by Kruskal-Wallis Test if it did not have normal distribution. The logarithmic regression was used to test the relationship between fish size (*TL* and *W*) and the batch fecundity [31]. For all the tests, SPSS software v.21 was used by setting a 5% significant level.

## Results

### Variation of pH and salinity

pH values did not have normal distribution (Shapiro-Wilk Test, NJ = 0.96, *p* < 0.01), but the reverse case was found in salinity (NJ = 0.99, *p* > 0.10). Table 1 illustrates the variation of pH and salinity monthly at six sampling sites. The pH values were not different from July 2019 to Jun 2020 but fluctuated around a narrow range from 7.73 ± 0.03 SE to 7.93 ± 0.04 SE (Kruskal-Wallis H, *χ*^*2*^ = 11.95, *p =* 0.37). Conversely, salinity expressed a monthly difference, reaching the highest values in February 2020 (25.83 ± 2.01 SE) and March 2020 (26.17 ± 2.06 SE, One-way ANOVA, *F* = 4.92, *p* < 0.01). Both pH and salinity varied among six sampling sites (*χ*^*2*^_*pH*_ = 29.48, *χ*^*2*^_*salinity*_ = 30.64, *p* < 0.01). pH in Hoa Binh (7.66 ± 0.05 SE) and Dam Doi (7.63 ± 0.05 SE) was lower than the four remaining places. Dong Hai and Dam Doi got the highest salinity of 23.50 ± 1.48 SE and 23.17 ± 1.21 SE. (Raw data can find in Raw data_pH and salinity file-supplementary).

**Table 1:** The variation of pH and salinity monthly at six sampling sites

Sampling sites	pH	Salinity
DHTV	7.85 ± 0.02^b^	12.33 ± 2.51^ab^
CLDST	7.81 ± 0.02^ab^	10.42 ± 1.47^a^
TDST	7.96 ± 0.06^b^	14.00 ± 2.06^ab^
HBBL	7.66 ± 0.05^a^	18.58 ± 1.47^bc^
DHBL	7.81 ± 0.03^ab^	23.50 ± 1.48^c^
DDCM	7.63 ± 0.05^a^	23.17 ± 1.21^c^
Shapiro-Wilk test	NJ = 0.96, *p* < 0.01	*NJ =* 0.99, *p >* 0.10
The Levene’s Test		*F =* 2.26, *p* = 0.06
One-way ANOVA		*F* = 10.16, *p* = 0.02
Kruskal-Wallis H Test	*χ*^*2*^ = 29.48, *p <* 0.01	
Month	pH	Salinity
Jul-19	7.78 ± 0.03	12.83 ± 2.65^a^
Aug-19	7.87 ± 0.03	11.33 ± 2.80^a^
Sep-19	7.93 ± 0.04	11.17 ± 2.51^a^
Oct-19	7.77 ± 0.19	11.83 ± 2.39^a^
Nov-19	7.73 ± 0.10	13.00 ± 2.68^a^
Dec-19	7.77 ± 0.08	15.33 ± 3.07^ab^
Jan-20	7.78 ± 0.03	21.83 ± 2.60^ab^
Feb-19	7.77 ± 0.04	25.83 ± 2.01^b^
Mar-19	7.73 ± 0.03	26.17 ± 2.06^b^
Apr-20	7.78 ± 0.05	22.00 ± 2.07^ab^
May-20	7.80 ± 0.04	17.33 ± 2.62^ab^
Jun-20	7.72 ± 0.07	15.33 ± 2.50^ab^
The Levene’s Test		*F* = 0.62, *p* = 0.81
One-way ANOVA		*F* = 0.80, *p* = 0.00
Kruskal-Wallis H Test	*χ*^*2*^ = 11.95, *p = 0.37*	

*DHTV* Duyen Hai, Tra Vinh, *CLDST* Cu Lao Dung, Soc Trang, *TDST* Tran De, Soc Trang, *HBBL* Hoa Binh, Bac Lieu, *DHBL* Dong Hai, Bac Lieu, *DDCM* Dam Doi, Ca Ma. The difference letters (a and b) in each category showed a significant difference

### Oogenesis

**Fig. 2:**
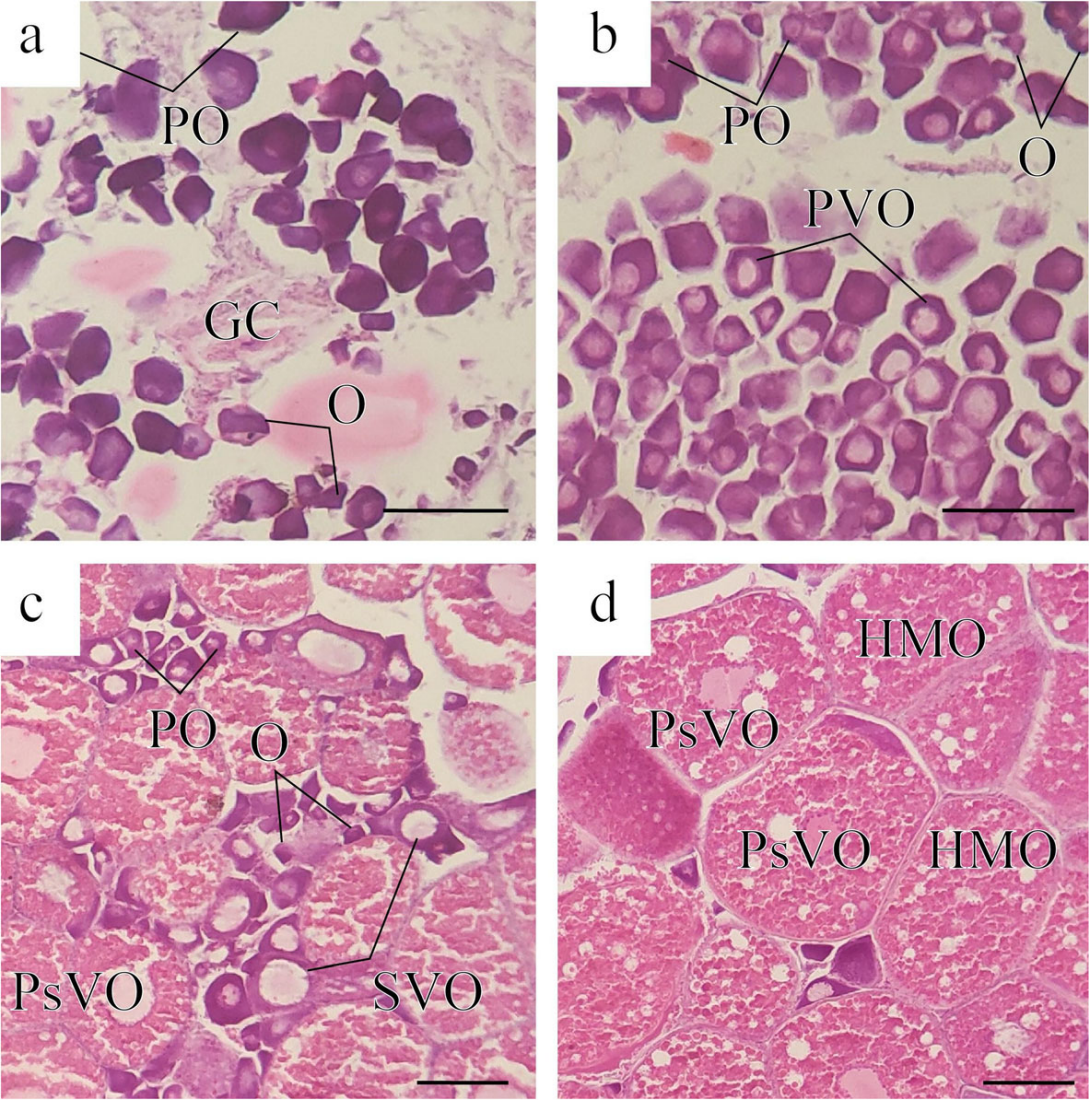
Histology of ovaries of *Butis koilomatodon* (**a**, **b**, **c**, and **d** labelled for histology of ovarian stages I, II, III, and IV, respectively). Ovarian sections show oogonia (O), germ cell (GC), primary oocyte (PO), primary vitellogenic oocytes (PVO), secondary vitellogenic oocytes (SVO), post vitellogenic oocytes (PsVO), hydrated oocytes (HMO). Scale bar: 50 μm for histology

Figure 2 illustrates the microscopic development stages of *Butis koilomatodon* ovaries. In the early growing stage I, the ovarian diameter was 0.82 ± 0.10 mm, thin and flat double strands in pale white color. Its surface was smooth and had several folds. It contained primary oocytes (PO) and oogonia (O), excluding germ cells (GC) (Fig. 2a). Oogonia were small and dark purple when being stained with hematoxylin. Its cytoplasm was observed. O was dominant in stage I. Primary oocytes (PO) were usually more extensive than O and could observe the nucleus because their color was lighter than the cytoplasm. The yolk was not formed in this period. In the growing stage or stage II, the ovaries’ diameter was 1.82 ± 0.11 mm, which was twice in size than stage I. It consists of O, PO, and primary vitellogenic oocytes (PVO) with some yolk granules in the cytoplasm (Fig. 2b). The ovary was yellowish and smooth with protruding blood vessels. Some oocytes snowballed into the oil-droplet stage in order to be seen inside the cytoplasm. Being accumulated in the cytoplasm during the maturing stage or stage III, the yolk granules and oil droplets had more eosin-basophilic (Fig. 2c). The size of the oocytes increased gradually with a 2.20 ± 0.18 mm diameter. The oocytes were granular; however, it was hard to separate them because of their tight linkage. O, PO, PVO, and secondary vitellogenic oocytes (SVO) with nucleus and yolk accumulation were found in the ovary, which became see-through with visible small yellowish eggs. PVO and SVO were more primary than O and PO. In the ripe and spawning stage or stage IV, the ovary consisted of most post vitellogenic oocytes (PsVO) and hydrated oocytes (HMO) besides some PO, PVO, and SVO (Fig. 2d). The ovaries continuously increased to the most significant size, with 4.07 ± 0.19 mm diameter. They were long, dark yellow, and smooth on the surface. Besides, they were swollen with prominent blood vessels. The eggs, which were easily separated from each other, were round, yellow, and visible by the naked eye. The oil droplets intermixed with yolk granules and became partially homogeneous. The nucleus shrank, and the nuclear membrane disappeared. The nucleolus migrated to the center of the nucleus, and then it was found easily in PsVO, but not in HMO. The ovaries in the degenerating stage (V) and the recovery stage (VI) were not found in the present study.

### Spermatogenesis

**Fig. 3:**
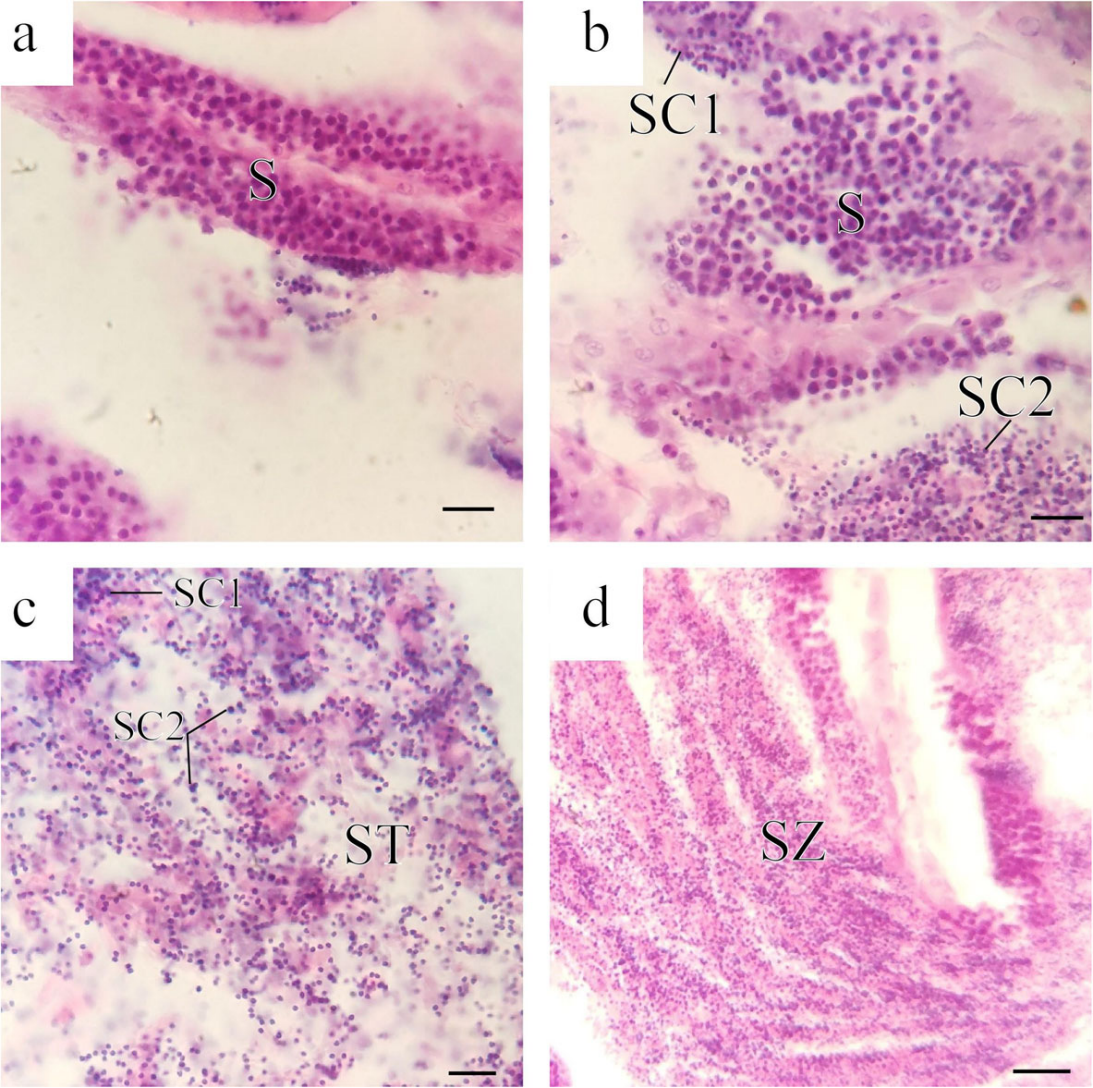
Histology of testis of *Butis koilomatodon* (**a**, **b**, **c**, and **d** labelled for histology of testis stages I, II, III, and IV, respectively). Testis sections show spermatogonia (S), primary spermatocytes (SC1), secondary spermatocytes (SC2), spermatid (ST), and spermatozoa (SZ). Scale bar: 10 μm for histology

During the early growing stage (stage I), testes mainly consisted of spermatogonia (S), which were scattered inside them (Fig. 3a). The diameter of the testes was 0.45 ± 0.05 mm (mean ± SD). Testes with an elongated shape and smooth surface were milky and easily confused with ovaries in stage I. The S was basophilic, having the dark purple color of hematoxylin. In the growing stage II, there were mainly primary spermatocytes (SC1), secondary spermatocytes (SC2), and a few S (Fig. 3b) in the cross-sectional histological specimen of testes. The testes’ diameter gradually increased to 0.72 ± 0.06 mm, and they were milky white, long, and slender. The dyed color of the nucleus in SC1 was darker than that in SC2. In the maturing stage III, a large number of spermatids (ST) and a small number of SC1 and SC2 were formed in the testicular lobules (Fig. 3c).

Testes continued to proliferate in their size (1.73 ± 0.07 mm in diameter). They were elongated, light yellow, and had a smooth surface and the sperm ducts appeared obviously. Besides ST and a few SC2, a group of spermatozoa (SZ) was produced in the testicular cavity and sperm ducts in the ripe stage and spermiation stage IV (Fig. 3d). During this stage, testicular lobules expanded and contained full of sperms. Testes were light yellow (slightly darker than they were in stage III). They were smooth, swollen, and were covered by blood vessels. They reached the most significant size at 2.77 ± 0.18 mm in diameter. The SZ was tiny cells with a round nucleus that had the dark color of hematoxylin. The mature males released their SZ during this stage. The degenerating stage V and the recovery stage VI were not obtained in this research.

### Gonadosomatic index, frequency of gonadal appearance and spawning season

Specimens of *Butis koilomatodon* (903 males and 411 females) were monthly collected at six sampling sites with the largest sample size in Hoa Binh (315 individuals) and the lowest one in Cu Lao Dung (112 individuals, Table 2; raw data can find in Raw data_*Butis koilomatodon* file-supplementary). In most of cases, Table 2 shows that the number of males was always higher than females with a sex ratio of 2.20:1.00.

**Table 2:** Number of *Butis koilomatodon* collected from six studied sites

Sampling time	DHTV		CLDST		TDST		HBBL		DHBL		DDCM	
	Male	Female	Male	Female	Male	Female	Male	Female	Male	Female	Male	Female
Jul-19	6	9	3	7	9	3	24	6	19	3	16	4
Aug-19	10	4	6	4	9	3	24	6	12	8	11	5
Sep-19	8	2	5	4	9	3	24	6	11	5	14	5
Oct-19	8	3	5	3	7	6	21	7	14	4	19	2
Nov-19	7	2	3	3	15	2	21	9	16	4	7	9
Dec-19	9	3	7	4	7	2	23	8	7	13	11	13
Jan-20	12	5	3	2	4	6	28	2	15	2	15	15
Feb-19	8	11	6	2	2	3	16	7	14	4	20	10
Mar-19	9	4	11	2	3	4	20	6	13	3	24	6
Apr-20	19	2	3	6	12	3	7	25	17	4	21	6
May-20	9	8	10	3	8	10	22	9	12	11	19	9
Jun-20	26	5	6	4	8	6	25	5	14	3	15	14
Sum	131	58	68	44	93	51	255	96	164	64	192	98

*DHTV* Duyen Hai, Tra Vinh, *CLDST* Cu Lao Dung, Soc Trang, *TDST* Tran De, Soc Trang, *HBBL* Hoa Binh, Bac Lieu, *DHBL* Dong Hai, Bac Lieu, *DDCM* Dam Doi, Ca Mau

**Fig. 4:**
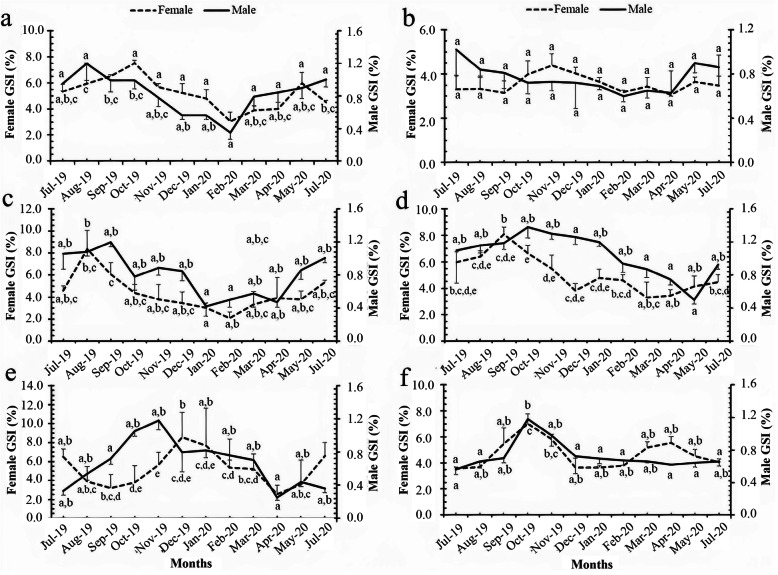
Monthly variation of gonadosomatic index of male and female *Butis koilomatodon* in Duyen Hai – Tra Vinh (**a**), Cu Lao Dung – Soc Trang (**b**), Tran De – Soc Trang (**c**), Hoa Binh – Bac Lieu (**d**), Dong Hai – Bac Lieu (**e**) and Dam Doi – Ca Mau (**f**)

*GSI* did not have a normal distribution (Shapiro-Wilk Test, NJ = 0.87, *p* < 0.01). *GSI* values varied between gender and season (Mann-Whitney U, *Z*_*gender*_ = 27.04, *Z*_*season*_ = 6.17, *p* < 0.01). The variation of gonadosomatic index (*GSI*) shows that values in the wet season (June–December) were higher than the dry season (January–May) for both genders in six study regions (Fig. 4). The alteration of GSI was also recorded amongst six sampling sites (Kruskal-Wallis H, *χ*^*2*^ = 40.72, *p <* 0.01).

**Fig. 5:**
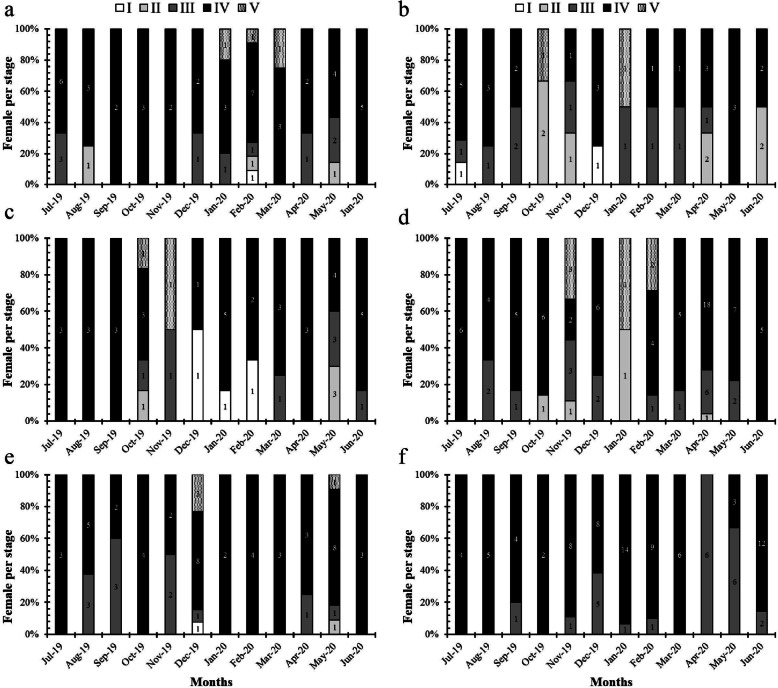
Ovaries stages compositions of *Butis koilomatodon* in Duyen Hai – Tra Vinh (**a**), Cu Lao Dung – Soc Trang (**b**), Tran De – Soc Trang (**c**), Hoa Binh – Bac Lieu (**d**), Dong Hai – Bac Lieu (**e**) and Dam Doi – Ca Mau (**f**)

**Fig. 6:**
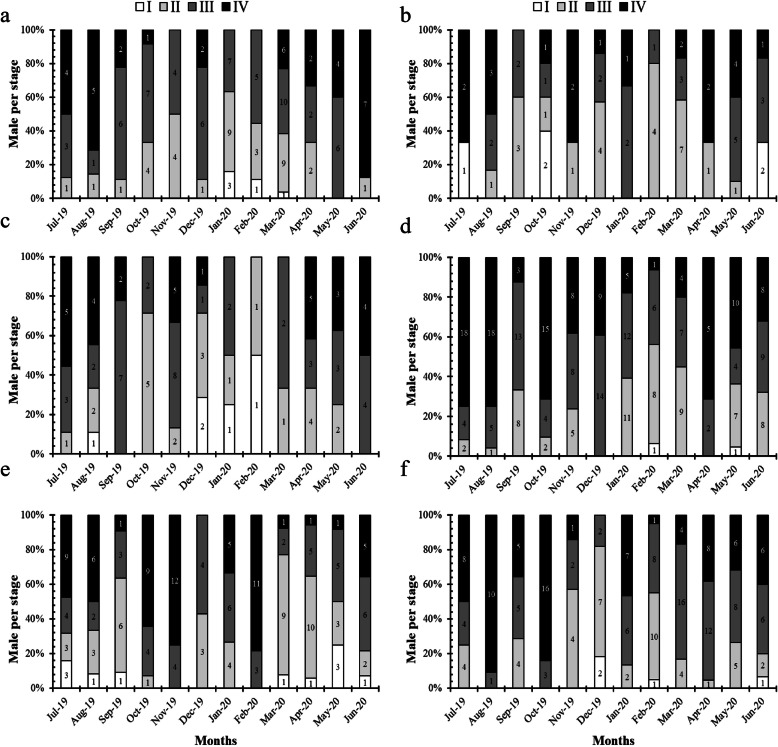
Testes stages compositions of *Butis koilomatodon* in Duyen Hai – Tra Vinh (**a**), Cu Lao Dung – Soc Trang (**b**), Tran De – Soc Trang (**c**), Hoa Binh – Bac Lieu (**d**), Dong Hai – Bac Lieu (**e**) and Dam Doi – Ca Mau (**f**)

Concerning sampling sites, the high *GSI* of males and females were recorded in Hoa Binh (1.07 ± 0.03 and 4.75 ± 0.25, respectively) (Kruskal-Wallis H, *χ*^*2*^_*male*_ = 141.58, *χ*^*2*^_*female*_ = 17.85, *p <* 0.01). There were significant differences between female and male *GSI* values at each of six sampling sites (Mann-Whitney U, *p <* 0.01 in all cases). Similar outcomes were found in *GSI* values of male and female monthly (Kruskal-Wallis H, *χ*^*2*^_*male*_ = 147.19, *χ*^*2*^_*female*_ = 29.74, *p <* 0.01). Specifically, *GSI* of females reached the highest mean values in August 2019 in Tran De (8.36 ± 1.7); in September 2019 in Hoa Binh (8.1 ± 0.51); in December 2019 in Dong Hai (8.57 ± 2.63); and in October in Dam Doi (7.01 ± 0.78). It was apparent that the highest female *GSI* fell into the wet season. The same trend was found in males’ *GSI* values, which peaked mostly in the wet season from August to November. For instance, male *GSI* reached the highest mean values in August 2019 in Duyen Hai (1.2 ± 0.21); in September 2019 in Tran De (1.2 ± 0.06); in October 2019 in Hoa Binh (1.38 ± 0.13); in November 2019 in Dong Hai (1.18 ± 0.11); and in October in Dam Doi (1.18 ± 0.06) Furthermore, mature males and females (stage IV) appeared almost every month (Figs. 5 and 6). Hence, it suggests that *Butis koilomatodon* spawned monthly in six habitats but mainly during the wet season.

From the histological observation of gonadal development and some of its unique characteristics, the cycle of reproduction was divided into several stages: stage I (early growing stage), stage II (growing stage), stage III (maturing stage), and stage IV (mature stage) [20]. Stage IV of the testes and ovaries were recorded almost all months in the study period (Figs. 5 and 6). Besides, the combination of the frequency of stage IV’s appearance and *GSI* values (Fig. 4) prove that this species displayed iteroparity throughout a year-round cycle. This conclusion was justified in the *GSI* data of Fig. 4. For example, in Dam Doi, both male and female *GSI* reach the highest values in October 2019. In other cases, almost high *GSI* values fell into the range from August to December 2019.

### Length at first mature and fecundity

**Fig. 7:**
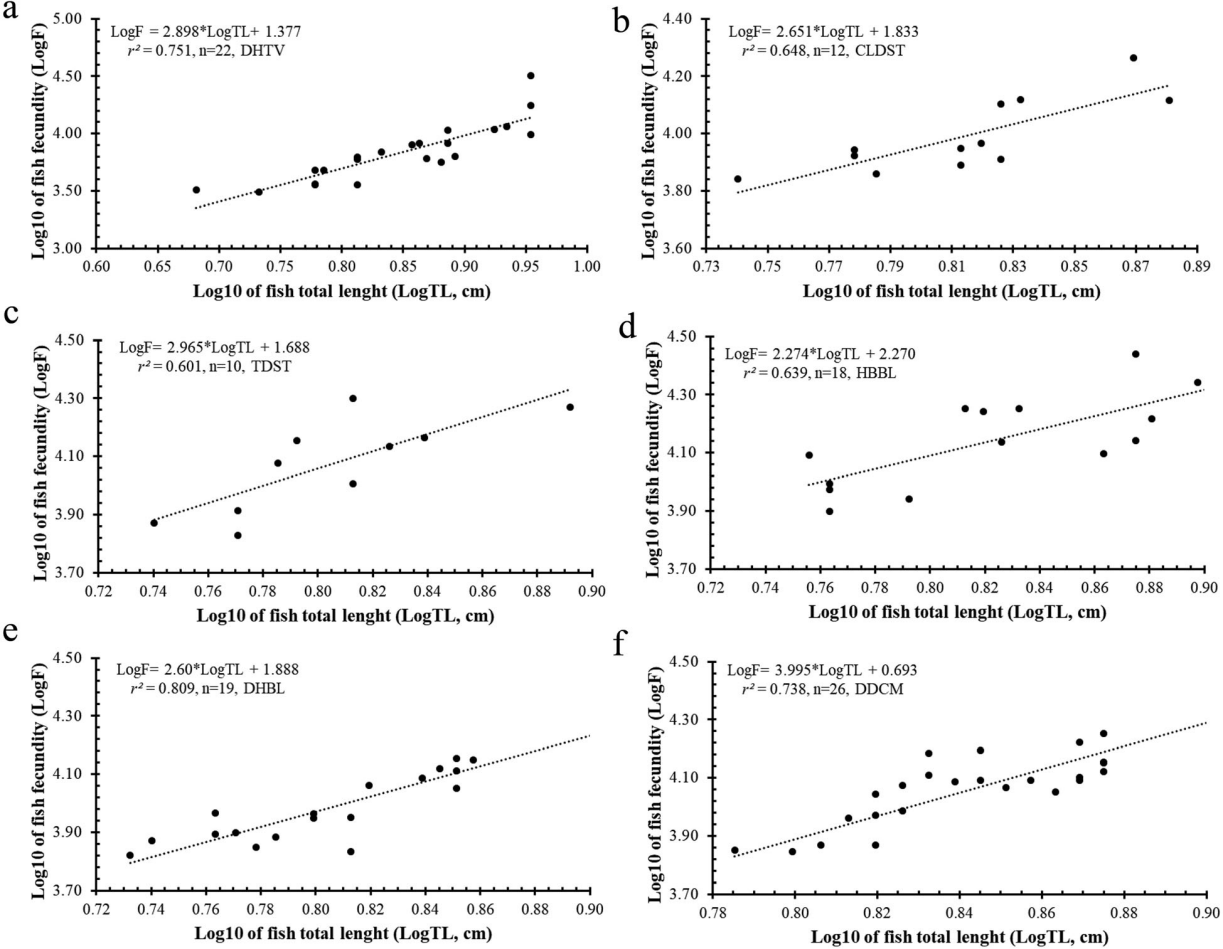
Relationships between fecundity and fish total length of *Butis koilomatodon* in Duyen Hai – Tra Vinh (**a**), Cu Lao Dung – Soc Trang (**b**), Tran De – Soc Trang (**c**), Hoa Binh – Bac Lieu (**d**), Dong Hai – Bac Lieu (**e**) and Dam Doi – Ca Mau (**f**)

**Fig. 8:**
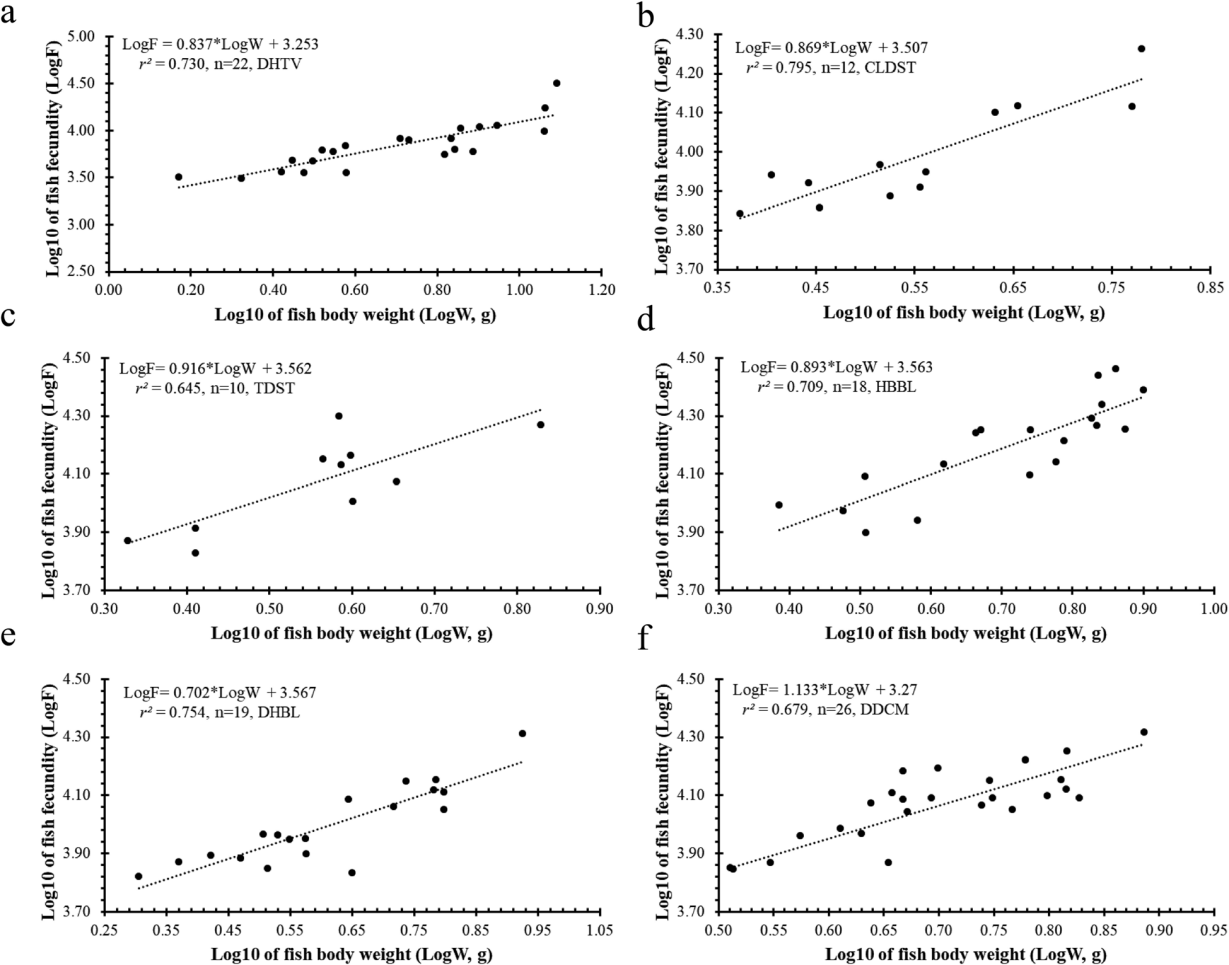
Relationships between fecundity and fish body weight of *Butis koilomatodon* in Duyen Hai – Tra Vinh (**a**), Cu Lao Dung – Soc Trang (**b**), Tran De – Soc Trang (**c**), Hoa Binh – Bac Lieu (**d**), Dong Hai – Bac Lieu (**e**) and Dam Doi – Ca Mau (**f**)

The length at first mature (*L*_*m*_) of *Butis koilomatodon* varied among their different habitats (Table 3). *L*_*m*_ of male and female ranged from 5.09 to 8.60 cm and 4.80 to 6.70 cm, respectively. The highest *L*_*m*_ values of both males and females were recorded at CLDST (male: 8.60 cm, female: 6.70 cm). Batch fecundity did not have a normal distribution (Shapiro-Wilk Test, NJ = 0.96, *p* < 0.01) and varied in the study sites and fluctuated from 3085 to 32,087 (Kruskal-Wallis H, *χ*^*2*^ = 35.55, *p <* 0.01, Table 3). The statistical result basing on average of fecundity at six sampling sites reveals that the highest fecundity value was recorded in Hoa Binh, Bac Lieu (16,686 ± 1396) and the lowest values in Duyen Hai (8217.23 ± 1354), Cu Lao Dung (10,207.17 ± 980), and Dong Hai (10,413.79 ± 813). Figure 7 illustrates that fecundity had a positive correlation to the female body length and can be inferred from the linear regression between the batch fecundity and the fish length. Besides, the fecundity of *Butis koilomatodon* was positively correlated fish weight (Fig. 8) due to the high of *r*^*2*^ value (> 0.6 for all cases).

**Table 3:** The length first mature stage and fecundity of *Butis koilomatodon* at six sampling sites

Category		DHTV		CLDST		TDST		HBBL		DHBL		DDCM		The Levene’s Test		One-way ANOVA	
		Male	Female	Male	Female	Male	Female	Male	Female	Male	Female	Male	Female	*F*	*p*	*F*	*p*
Length at first mature		7.00	4.80	8.60	6.70	6.93	5.50	5.09	5.40	5.10	5.50	5.30	4.90				
Fecundity (number of eggs)	Range	3085-32,087		6958-18,343		6731-19,986		7939-28,955		6632-20,588		4704-20,813		1.29	0.27	6.88	< 0.01
	Mean ± SD	8217 ± 1354^a^		10,207 ± 980^a^		12,547 ± 1433^ab^		16,686 ± 1396^b^		10,413 ± 813^a^		11,947 ± 719^ab^					

*DHTV* Duyen Hai, Tra Vinh, *CLDST* Cu Lao Dung, Soc Trang, *TDST* Tran De, Soc Trang, *HBBL* Hoa Binh, Bac Lieu, *DHBL* Dong Hai, Bac Lieu, *DDCM* Dam Doi, Ca Mau. The difference letters (a and b) in each category showed a significant difference

## Discussion

Swennen et al. [32] noted that some goby species could adapt well to a different water body with a wide range of salinity up to 25‰ and temperature from 28.7 to 36.8 °C. Indeed, *Butis koilomatodon* in the present study was found in all six sites in both dry and wet seasons with 7.72–7.93 pH and 11.17–26.17‰ salinity. However, some gobies’ appearance depended on salinity change; for example, *Butis butis* was only found in Soc Trang in December and February 2013 while the salinity increased sharply from 2‰ to 12‰ [33].

In the present study, the histological analysis showed that the spawning season of *Butis koilomatodon* lasted throughout a year since ripe ovaries and testis (stage IV) appeared every month in all research regions. This breeding model was found in *Butis butis* with mature testes and ovaries appeared monthly [25], and *Valenciennea strigata* kept laying eggs every 13 days for all-year-round [34]. Contrary, *Tridentiger trigonocephalus* in the Southern Coastal Waters of Korea started spawning from May and peaking in the June–July period known as spring-summer spawners [35]. The spring-summer spawning was detected in the striped goby *Acentrogobius pflaumi* spawning in the May–June period [36], *Chasmichthys dolichognathus* reproducing in the April–July phase [37], and *Tridentiger obscurus* depositing in the June–July time [38]. The contrast in temperature between winter and summer seasons in Korea could lead to this difference. Some of the species would breed when nutrient was abundant in the wet season. For example, in Imo estuary, Nigeria, *Periophthalmus barbarus* spawned mainly in May [39]; in Malaysia’s coastal mudflat, the reproductive season of *Periophthalmodon schlosseri* was from April to October [40]. Several of gobies living in the estuarine in the Mekong Delta also spawned in the wet season due to nutrient richness in the dry season, namely *Pseudaporcyptes elongatus* [41], *Boleophthalmus boddarti* [42], *Parapocryptes serperaster* [8], *Trypauchen vagina* [43], and *Stigmatogobius pleurostigma* [44].

According to Nedeco [45], Mekong Delta - a vastly alluvial plain supplies available habitat, food source, and spawning ground for the different fish species, including goby. The changes in *GSI* had a close relation with reproductive behavior. The *GSI* value was higher in months when fish spawned. Combining the appearance of ovaries and testes stages IV for every month and the value of *GSI* suggests that *Butis koilomatodon* bred monthly in in situ each habitat. Hence, they were able to adapt well to the changing conditional factors, and their distribution was widespread. The opposite results were found in *Pseudapocryptes elongatus* [41], *Boleophthalmus boddarti* [42], *Parapocrytpes serperaster* [8], *Trypauchen vagina* [43], and *Stigmatogobius pleurostigma* [44]. These gobies co-live *Butis koilomatodon* in estuarine regions of Mekong Delta, but their reproductive season was mainly in the wet season.

The findings of many different oocytes in the mature ovary prove that *Butis koilomatodon* in estuaries regions in the Mekong Delta was a multiple spawner, like most other gobies [7]. The species *Butis koilomatodon* could release SZ many times during the spawning period as different spermatocytes (SC1, SC2, ST, and SZ) were found in the stage IV testes. A similar pattern was found in other gobies co-living in Vietnam for example, *Pseudapocryptes elongatus* [41]; *Boleophthalmus boddarti* [42]; *Parapocryptes serperaster* [8]; *Trypauchen vagina* [43] and *Stigmatogobius pleurostigma* [44]. *Periophthalmodon septemradiatus* deposited eggs monthly throughout the year in some provinces in the Mekong Delta [46]. A multiple spawner was also found in *Butis butis,* a neighbor species of the goby in the present study, according to Dinh and Le [25]. Other gobies living outside of Vietnam were also multiple spawner such as *Valenciennea strigata* in French Polynesia [34]; *Neogobius melanostomus* in North America [47]; *Periophthalmus barbarus* in Japan [39]; and *Amblygobius phalaena* in Nigeria [48]. The reproductive strategy and behavior of *Butis koilomatodon* were similar among six sampling sites, indicating that the spatial factor might not affect its breeding pattern.

The length at first mature (*L*_*m*_) is considered to be a specific characteristic for goby species and related to an egg caring tendency; for example, male *Pomatoschistus marmoratus* took care of the fertilized eggs, leading to the longer *Lm* in males than females [49]. It was easier to compare when using the *L*_*m*_*/TL*_*max*_ ratio instead of *L*_*m*_ values because of the difference in each fish species’ total length. *Butis koilomatodon* shows the same tendency to protect eggs, supported by the length at first mature of males was longer than females. The similar behavior found in other gobies with *L*_*m*_*/TL*_*max*_ was higher than *Butis koilomatodon* (*L*_*m*_*/TL*_*max*_ was 0.57–0.65) such as *Periophthalmus barbarus* with male and female *L*_*m*_*/TL*_*max*_ ratios were 0.88 and 0.79 [39]; *Pseudapocryptes elongatus* with male and female *L*_*m*_*/TL*_*max*_ ratios were 0.73 and 0.69 [41]; *Stigmatogobius pleurostigma* with female *L*_*m*_*/TL*_*max*_ proportion was 0.73 [44]. By contrast, the result found in Dinh and Le [25] shows that *L*_*m*_*/TL*_*max*_ ratio of *Butis butis* was lower than *Butis koilomatodon* (0.57 for male and 0.43 for female). In Hoa Binh and Dong Hai, Bac Lieu, the males’ *L*_*m*_ was slightly shorter than females because of differences in the environmental factors (the ratio *L*_*m*_/*TL*_*max*_ of male and female were 0.62 and 0.66, respectively). The length at the first mature of females was found higher than that of males in *Trypauchen vagina* (*L*_*m*_*/TL*_*max*_ of males and females were 0.83 and 0.84) [43].

According to McDowall [50], the higher fecundity can recover the population despite the higher larval mortality caused by unpredictable environmental factors. Moreover, Ha and Kinzie [14] note that gobiids’ fecundity changed greatly from one species to another, with the lowest of 100 eggs in *Eviota lacrimae* and the highest of ~ 500,000 eggs in *Awaous guamensis*. Lehtonen et al. [51] state that salinity greatly influenced the fish distribution and population structure. Nevertheless, the effects of salinity on the reproduction of fish were still not well known. In this present study, the batch fecundity (F) of *Butis koilomatodon* (*F* = 3085–32,087) was different between habitats and was not impacted by the salinity of the water. Although the salinity was significantly different between six sampling sites, there was no clear relationship between salinity and fecundity. So, it can be inferred that the fecundity of this mud sleeper was affected by spatial factors and the condition of the female body (length and weight). It is clear that in *Boleophthalmus boddarti*, their batch fecundity in India ranged 2100–12,300 eggs [52], and the number was higher in Vietnam 9800–33,800 eggs [42] despite the environmental pollution. The different goby species has different fecundity, but most of their fecundity increases when the body length and weight increase [53]. Similarly, this correlation was found positively in some gobies co-habitat in Mekong delta such as *Pseudapocryptes elongatus* (*F* = 2652–29,406) [41], *Boleophthalmus boddarti* (*F* = 9800–33,800) [42], *Parapocryptes serperaster* (*F* = 6000–11,700) [8], *Butis butis* (*F* = 15,000–78,500) [25], *Trypauchen vagina* (*F* = 4000–12,750) [43], *Stigmatogobius pleurostigma* (*F* = 3100–5650) [44] and *Periophthalmodon septemradiatus* (*F* = 969–17,536) [46]. Several gobiids species living outside of Vietnam also show a positive relationship between fecundity and fish sizes, including *Valenciennea strigata* (*F* = 60,000–160,000) [34] *Neogobius melanostomus* (*F* = 84–606) [47], *Crystallogobius linearis* (*F* = 200–700) [54], *Amblygobius phalaena* (*F* = 37,000–38,000) [48] and *Periophthalmus barbarus* (*F* = 900–24,000) [39].

## Conclusion

The gonads histological and the *GSI* monthly variation indicates that *Butis koilomatodon* can be considered a multiple spawner with indeterminated fecundity throughout the year. Their fecundity was positively correlated to the increase in fish size (TL and W) and varied with different regions. The length at first maturity of *Butis koilomatodon* male was longer than female. It is important for fishery and local authorities to manage the fishing activities and conserve the fish population, such as choosing mesh size and time of year suitably to catch fish. This knowledge of reproductive traits gained from this study is a useful reference source for future studies and helps manage this species’ population.

## Supplementary Information

**Additional file 1.**

**Additional file 2.**

## Data Availability

The data used during the current study are available from the corresponding author on reasonable request.

## Abbreviations

*GSI*: Gonadosomatic index
PO: Primary oocytes
O: Oogonia
GC: Germ cells
PVO: Primary vitellogenic oocytes
SVO: Secondary vitellogenic oocytes
PsVO: Post vitellogenic oocytes
HMO: Hydrated oocytes
S: Spermatogonia
SC1: Primary spermatocytes
SC2: Secondary spermatocytes
ST: Spermatids
SZ: Spermatozoa
*L*_*m*_: The length of the first mature stage
*r*^*2*^: The determinative parameter
*F*: The batch fecundity
